# Supplementary material for: Drug repurposing for aging research using model organisms
Source: Aging Cell. 2017 Jun 16;16(5):1006–15. doi: 10.1111/acel.12626 (PMC5595691; doi:10.1111/acel.12626)
Supplement: Supplementary file 7 — Data S1 Zip‐Archive of all report cards. [file ACEL-16-1006-s007.zip › RC_URE.pdf]

URE

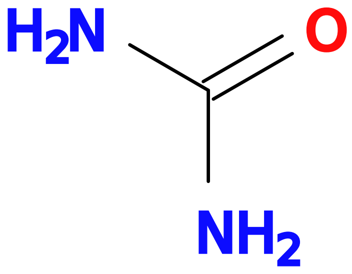

Database identifiers

ChEMBLCompound	CHEMBL985
DrugBank	DB03904
CHEBI	16199
CHEBI	48376
ZINC	ZINC08214514
eMolecules	10525132
eMolecules	475133

Ranking

	Rank	Score
Drosophila	311/697	0.514
C. elegans	NA	NA

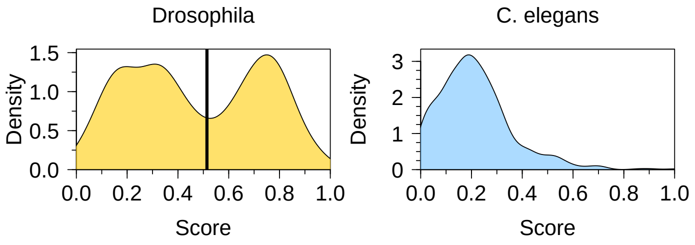

	Ageing implication	Domain conservation	Binding site conservation	Binding affinity	Bioavailability	Lipinski	Promiscuity	Purchasability	Drug approval	**Total**
Drosophila	0.624	0.966	0.883	0.658	(0.9)	0.0	-0.001	0.1	0.1	0.514
C. elegans	NA	NA	NA	NA	NA	NA	NA	NA	NA	NA

Names

- **carbamimidic acid**
- carbamimic acid
- carbonamidimidic acid
- E927b
- H2n-c(=nh)-oh
- H2n-c(oh)=nh
- Ho-c(=nh)-nh2
- Isoharnstoff
- isourea
- pseudourea
- Urea
- Ureaphil

Roles

ChEBI entry 48376 has no roles

Status

Approved drug (according to ChEMBL)	Yes
Number of Rule of 5 violations	0
Binding affinity to original target in log units (RF-Score prediction)	5.66
Burns *C. elegans* bioavailability prediction	-2.09

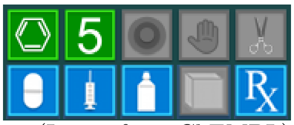

(Image from ChEMBL)

Medical Information

Retail: A. Aarons Inc., Aerosol Scandinavia Ab, Allan Pharmaceutical LLC, American Spraytech LLC, APL Logistics WMS, Avent Inc. and 43 more suppliers

(Information from DrugBank)

Compound Target Characteristics

Arginase-1

Best gene implication in ageing for this target family came from gene P07824 via mapping the annotation from RGD 2150 annotated in RGD 2014-03-11. Annotation GO 7568 (aging) was Inferred from Expression Pattern

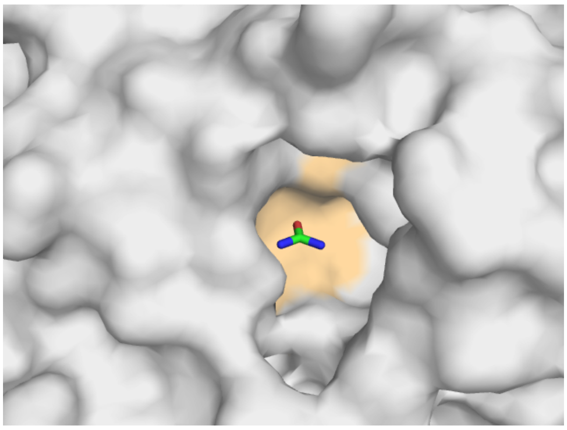

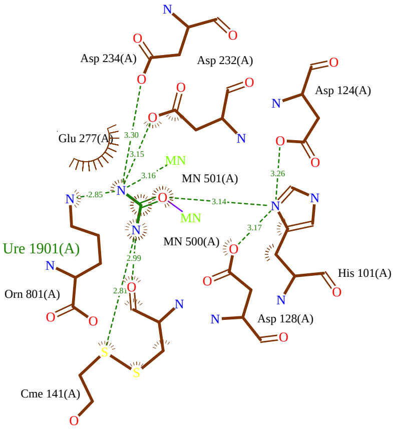

protein	amino acids contacts (binding site)
PDB:1hqg:chainA:P07824	H D D X D D E
sp:P05089:ARGI1_HUMAN	H D D H D D E
sp:P07824:ARGI1_RAT	H D D H D D E
sp:Q61176:ARGI1_MOUSE	H D D H D D E
tr:Q9NHA5:Q9NHA5_DROME	H D D H D D E
tr:O76895:O76895_DROME	H D D H D D E
sp:P00812:ARGI_YEAST	H D D H D D E

	whole protein		domain-based		contact-based	
protein	ident	simil	ident	simil	ident	simil
PDB:1hqg:chainA:P07824	1.0	1.0	1.0	1.0	1.0	0.85
sp:P05089:ARGI1_HUMAN	0.87	0.96	0.87	0.96	0.86	0.88
sp:P07824:ARGI1_RAT	1.0	1.0	1.0	1.0	0.86	0.88
sp:Q61176:ARGI1_MOUSE	0.94	0.98	0.93	0.98	0.86	0.88
tr:Q9NHA5:Q9NHA5_DROME	0.33	0.71	0.35	0.74	0.86	0.88
tr:O76895:O76895_DROME	0.33	0.7	0.35	0.74	0.86	0.88
sp:P00812:ARGI_YEAST	0.37	0.71	0.38	0.72	0.86	0.88

arg (FBgn0023535) associated phenotypes
partially lethal - majority die, some die during pupal stage

(Information from FlyBase)

arg (UniProt:Q9NHA5) annotation
Cofactor: Mn(2+) Evidence=(RuleBase:RU361159); Note=Binds 2 manganese ions per subunit. (RuleBase:RU361159);
Pathway: Nitrogen metabolism; urea cycle; L-ornithine and urea from L-arginine: step 1/1. (RuleBase:RU361159).

(Information from UniProt)

arg (UniProt:O76895) annotation
Cofactor: Mn(2+) Evidence=(RuleBase:RU361159); Note=Binds 2 manganese ions per subunit. (RuleBase:RU361159);
Pathway: Nitrogen metabolism; urea cycle; L-ornithine and urea from L-arginine: step 1/1. (RuleBase:RU361159).

(Information from UniProt)

Catenin beta-1

Best gene implication in ageing for this target family came from gene B4DGU4 via mapping the annotation from Ensembl ENSG00000168036 via mapping the annotation from EntrezGene 1499 via mapping the annotation from GenAgeHuman 0223 annotated in GenAge release 17.

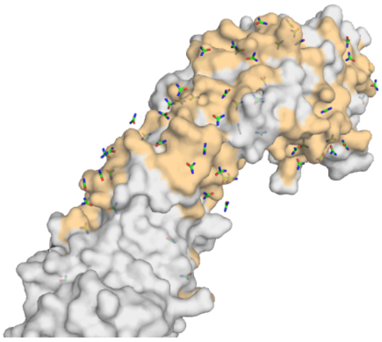

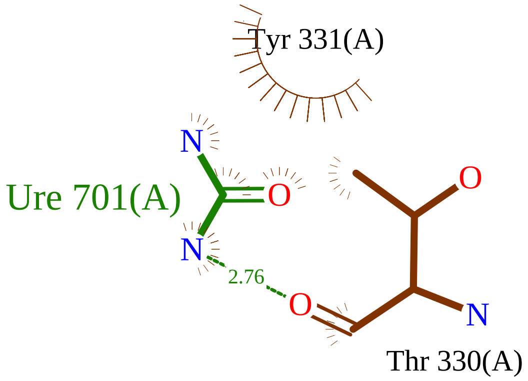

protein	amino acids contacts (binding site)
PDB:3bct:chainA:Q02248	- - Q N K M L G S D S L F Y L L Q E G A K R L G Q A L L N K T N V K F L Q Y G
PDB:4ev8:chainA:Q02248	H K Q N K M L G S D S L F Y L L Q E G A K R L G Q A L L N K T N V K F L Q Y G
PDB:4ev9:chainA:Q02248	H K Q N K M L G S D S L F Y L L Q E G A K R L G Q A L L N K T N V K F L Q Y G
PDB:4eva:chainA:Q02248	H K Q N K M L G S D S L F Y L L Q E G A K R L G Q A L L N K T N V K F L Q Y G
PDB:4evp:chainA:Q02248	H K Q N K M L G S D S L F Y L L Q E G A K R L G Q A L L N K T N V K F L Q Y G
PDB:4evt:chainA:Q02248	H K Q N K M L G S D S L F Y L L Q E G A K R L G Q A L L N K T N V K F L Q Y G
tr:B4DGU4:B4DGU4_HUMAN	H K Q N K M L G S D S L F Y L L Q E G A K R L G Q A L L N K T N V K F L Q Y G
sp:P35222:CTNB1_HUMAN	H K Q N K M L G S D S L F Y L L Q E G A K R L G Q A L L N K T N V K F L Q Y G
sp:Q9WU82:CTNB1_RAT	H K Q N K M L G S D S L F Y L L Q E G A K R L G Q A L L N K T N V K F L Q Y G
sp:Q02248:CTNB1_MOUSE	H K Q N K M L G S D S L F Y L L Q E G A K R L G Q A L L N K T N V K F L Q Y G
sp:P18824:ARM_DROME	H K S N K L L S S E S L F Y L L Q D G S K R L G Q T L L Q R N N V K F L Q Y G

protein	whole protein ident	simil	domain-based ident	simil	contact-based ident	simil
PDB:3bct:chainA:Q02248	1.0	1.0	1.0	1.0	0.99	0.97
PDB:4ev8:chainA:Q02248	1.0	1.0	1.0	1.0	1.0	1.0
PDB:4ev9:chainA:Q02248	1.0	1.0	1.0	1.0	1.0	1.0
PDB:4eva:chainA:Q02248	1.0	1.0	1.0	1.0	1.0	1.0
PDB:4evp:chainA:Q02248	1.0	1.0	1.0	1.0	1.0	1.0
PDB:4evt:chainA:Q02248	1.0	1.0	1.0	1.0	1.0	1.0
tr:B4DGU4:B4DGU4_HUMAN	0.99	0.99	1.0	1.0	1.0	1.0
sp:P35222:CTNB1_HUMAN	1.0	1.0	1.0	1.0	1.0	1.0
sp:Q9WU82:CTNB1_RAT	1.0	1.0	1.0	1.0	1.0	1.0
sp:Q02248:CTNB1_MOUSE	1.0	1.0	1.0	1.0	1.0	1.0
sp:P18824:ARM_DROME	0.65	0.83	0.79	0.94	0.72	0.86

arm (FBgn0000117) associated phenotypes

RU486 conditional, cell autonomous, cell migration defective, cell non-autonomous, cell polarity defective, cell shape defective, germline clone, heat sensitive, hyperplasia, increased cell death, lethal - all die before end of pupal stage, memory defective, mitotic cell cycle defective, neuroanatomy defective, partially lethal - majority die, planar polarity defective, segment polarity phenotype, size defective, somatic clone, some die during pupal stage

(Information from FlyBase)

arm (UniProt:P18824) annotation

Function: Isoform neural may associate with CadN and participate in the transmission of developmental information. Can associate with alpha-catenin. Isoform cytoplasmic accumulates through wg signaling; arm function in wg signal transduction is required early in development for determination of neuroblast fate. Arm and Abl proteins function cooperatively at adherens junctions in both the CNS and epidermis. (PubMed:9635189).

Subunit: Interacts with Mer and Moe at the adherens junction. Interacts with Inx2. (PubMed:15047872, PubMed:8666669).

Subcellular location: Cytoplasm ECO:0000269—PubMed:8666669. Cell membrane (PubMed:8666669); Peripheral membrane protein (PubMed:8666669); Cytoplasmic side (PubMed:8666669). Cell junction, adherens junction Note=Inner surface of cell membrane and adherens junction.

Tissue specificity: Isoform cytoplasmic accumulates at low levels in axons, at high levels in specific cells along the CNS midline and in leg and eye imaginal disks. Isoform neural accumulates in the axon tracts of the CNS. Both isoforms accumulate in the peripheral nervous system. (PubMed:9635189).

Developmental stage: Present at all stages, but reaches the highest levels during early to mid-embryogenesis. Isoform cytoplasmic is the predominant one from the cellular blastoderm stage until germ-band retraction. Isoform neural is first seen after germ band retraction.

Ptm: Phosphorylated on Ser, Thr and Tyr residues. Level of phosphorylation varies both during embryonic development and from embryonic tissue to tissue. Sgg is required for phosphorylation and wg signal negatively regulates arm phosphorylation. Hypophosphorylated form of arm increases in steady-state levels. (PubMed:18327897, PubMed:7529201).

(Information from UniProt)
